# Correlation analysis between filamentous fungi and chemical compositions in a pu‐erh type tea after a long‐term storage

**DOI:** 10.1002/fsn3.1543

**Published:** 2020-04-13

**Authors:** Binxing Zhou, Cunqiang Ma, Xiaoying Ren, Tao Xia, Chengqin Zheng, Xiaohui Liu

**Affiliations:** ^1^ College of Long Run Pu‐erh Tea Yunnan Agricultural University Kunming China; ^2^ Kunming Dapu Tea CO., LTD Kunming China; ^3^ Liaocheng Senior Financial Vocational School Liaocheng China; ^4^ State Key Laboratory of Tea Plant Biology and Utilization Anhui Agricultural University Hefei China

**Keywords:** filamentous fungi, liquid chromatography, metabolites, storage, tea

## Abstract

Storage environment caused the difference between Jinhua Pu‐erh tea (JPT) and General Pu‐erh tea. In this study, fungal flora and chemical compositions were analyzed. The results showed that storage environment caused significant (*p* < .05) differences of theaflavins (TF), theabrownins (TB), tea polyphenols (TP), and water‐soluble sugars (WSS), and a highly significant (*p* < .01) difference of thearubigins (TR). *Aspergillus niger*, *Aspergillus pallidofulvus*, *Aspergillus sesamicola*, *Penicillium manginii,* and *Aspergillus tamarii* were isolated from Pu‐erh teas and identified based on colony characteristics and ITS, β‐tubulin, and calmodulin gene sequences, respectively. *A. pallidofulvus*, *A. sesamicola*, and *P. manginii* were dominant fungi in JPT and generated macroscopic yellow cleistothecia after a long‐term storage. Correlation analysis showed that dominant fungi exhibited significantly (*p* < .05 or *p* < .01) positive or negative corrections with TF, TB, TP, WSS, TR, and gallic acid. This study revealed dominant fungi including *A. pallidofulvus, A. sesamicola*, and *P. manginii* and their effects on given chemical compositions.

## Graphical abstract

*Aspergillus pallidofulvus*, *Aspergillus sesamicola*, and *Penicillium manginii* selected from Pu‐erh tea were dominant fungi, which could generate macroscopic yellow cleistothecia and have specific effects on given chemical compositions after a long‐term storage. This study revealed the dominant fungi and their effects on chemical compositions during the storage of Pu‐erh tea.

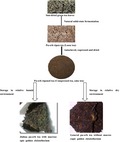

## 1 INTRODUCTION

Chinese dark tea, including Yunnan Pu‐erh tea, Hunan Fu‐zhuan tea, Hubei Qing‐zhuan tea, Sichuan Bian‐xiao tea, and Guangxi Liu‐bao tea, is a kind of microbial fermented tea in which fermentation allows microorganisms to induce auto‐oxidation and nonenzymatic auto‐oxidation to complete the process (Lv et al., 2014; Xu et al., 2011). Pu‐erh tea (Pu‐erh shucha), a traditional Chinese dark tea, has been produced in Yunnan Province of China with a production history of more than a thousand years (Peng et al., 2013). Based on recent researches, Pu‐erh tea has definite efficacy on reduction of waist fat buildup and atherosclerosis probability, antioxidation, antibiosis, anticancer, antidiabetics (Lee & Foo, 2013; Su, Wang, Song, Bai, & Li, 2016), hypolipidemic (Deng, Lin, Shyur, & Lin, 2015; Way et al., 2009), and antirheumatic (Lyu et al., 2013; Nyambe & Williamson, 2016).

Pu‐erh tea is normally made from sun‐dried green tea leaves (*Camellia sinensis* var. *assamica* (JW Masters) Kitamura) by microbial solid‐state fermentation (pile fermentation) at high temperature (about 50°C) and high humidity condition (Lv, Zhang, Lin, & Liang, 2013; Zhang et al., 2016). Due to the participation of microorganism, the storage of Pu‐erh tea could be regarded as postfermentation process (Gao, Bian, Mi, Hu, & Wu, 2016; Zhang, Li, Ma, & Tu, 2011). Pu‐erh tea could be stored in natural condition to make pu‐erh aged tea after a long‐term storage for years, and quality increases with aging time (Xu et al., 2019).

Although Pu‐erh tea has been processed, stored, and consumed for hundreds of years, its microbiological properties, particularly fungal community associated with Pu‐erh tea storage, are not completely understood. To date, just several actinomycetes and bacteria are detected in Pu‐erh tea storage, such as *Actinoplanes aurantiacus, Actinoplanes pallidoaurantiacus, Actinoplanes purpeobrunneus, Streptomyces bacillaris*, and *Streptomyces cinereus* (Chen, Chan, Chang, Liu, & Chen, 2009; Chen, Liu, & Chang, 2010). *Aspergillus* spp. and *Penicillium* spp. are dominant fungi found in pu‐erh tea storage (Tian, Zhu, Wu, Wang, & Liu, 2013).

The macroscopic yellow cleistothecia called “golden flowers” are traditionally judged as a quality standard of Fu‐zhuan tea (Li et al., 2019). Due to the difference in processing techniques, golden flowers were hardly found in solid‐state fermentation of pu‐erh tea. However, in a particular storage environment, the golden flowers similar to that in Fu‐zhuan tea could appear in Pu‐erh tea occasionally named as Jinhua pu‐erh tea (JPT) with higher sensory quality, which was chosen in this work to investigate the correlation between filamentous fungi and chemical compositions in the storage. In this paper, five filamentous fungal strains were isolated from pu‐erh tea and identified as *Aspergillus niger, Aspergillus pallidofulvus*, *Aspergillus sesamicola, Penicillium manginii,* and *Aspergillus tamari* based on ITS, β‐tubulin, and calmodulin gene sequences, respectively. *A. pallidofulvus*, *A. sesamicola,* and *P. manginii* were dominant fungi and generated macroscopic yellow cleistothecia after a long‐term storage in relative humid environment. Dominant fungi improved tea quality in aroma and taste, and had significant (*p* < .05) impacts on specific chemical compounds during the storage.

## 2 MATERIALS AND METHODS

### 2.1 Samples

**FIGURE 1:**
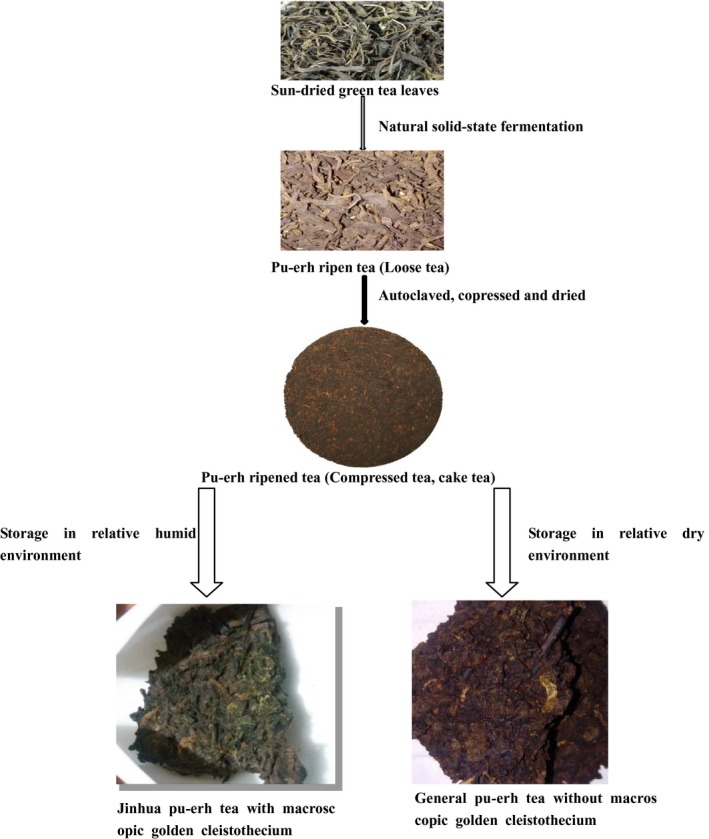
The processing and storage of pu‐erh tea type. Pu‐erh tea used in this paper was made from the same batch of sun‐dried green tea leaves with identical processing. Due to the different storage environments, the Pu‐erh tea used were divided into Jinhua Pu‐erh tea and General Pu‐erh tea according to appearance

Pu‐erh tea samples: JPT and General Pu‐erh tea (GPT) were processed and offered by Kunming Dapu Tea CO. Ltd, Yunnan Province, China. Both pu‐erh teas were made from identical pu‐erh ripen tea (loose tea) after they were autoclaved, compressed, and dried, and were stored at different natural conditions in Kunming, Yunnan Province, China. The processing and storage of Pu‐erh teas are shown in Figure 1. After a long‐term storage for about 15 years, macroscopic golden cleistothecia appeared on the surface and in the inside of the Pu‐erh tea stored at relative humid environment that was defined as JPT; GPT stored at relative dry environment was defined as control without visible golden flowers.

### 2.2 Chemicals and reagents

Standards of (+)‐catechin (C), (−)‐epicatechin (EC), (−)‐epigallocatechin (EGC), (−)‐epicatechin 3‐*O*‐gallate (ECG), (−)‐epigallocatechin 3‐*O*‐gallate (EGCG), gallic acid (GA), and caffeine were purchased from Sigma‐Aldrich Co. Ltd. SP fungal DNA Kit, DNA marker, polymerase chain reaction (PCR) spread reagent, and PCR primers, ITS1 (5′‐TCCGTAGGTGAACCTGCGG‐3′) and ITS4 (5′‐TCCTCCGCTTATTGATAGC‐3′); Bt2a (5′‐GGTAACCAAATCGGTGCTGCTTTC‐3′) and Bt2b (5′‐ACCCTCAGTGTAGTGACCCTTGGC‐3′); and CF1L (5′‐GCTGACTCGTTGACCGAAGAG‐3′) and CF4 (5′‐ATTTTTGCATCATGAGCTGAAC‐3′), were purchased from TaKaRa Biotechnology Co. Ltd. Acetonitrile, methanol, and acetic acid for high‐performance liquid chromatography (HPLC) were purchased from Mreda Biotechnology Co. Ltd.

### 2.3 Isolation and calculation of filamentous fungi

Tea samples were used to isolate the fungi using potato dextrose agar (PDA) medium, and they were counted by dilution‐plating method (Wang, Peng, & Gong, 2011). The colony‐forming units were calculated by per dry weight gram of tea leaves after cultivation at 30°C for 2 days (Wang, Gong, Chisti, & Sirisansaneeyakul, 2015).

### 2.4 Fungal identification

The colony morphological characteristics and conidia structure of isolated filamentous fungi were observed after cultivation on PDA medium and Czapek yeast extract agar medium at 25°C for 7 days (Zhou, Ma, Wang, & Xia, 2018). The filamentous fungus grew aerobically as pure cultures in 20 ml of Czapek Dox medium in 125‐ml shake flasks at 30°C, 250 rpm, for 2 days. Fresh cells were obtained by centrifugation at 1,700 ***g*** for 5 min and freeze‐dried at −80°C (Wang et al., 2015; Zhao et al., 2013). DNA was extracted by using SP Fungal DNA Kit. The fungal primers ITS1 and ITS4, Bt2a and Bt2b, and CF1L and CF4 were used in the PCR to amplify the ITS, β‐tubulin, and calmodulin regions, respectively (Abe et al., 2008). The final volume of 50 μl, containing 1.0 μl of template DNA, 5 μl of 10× buffer, 5 μl of dNTPs (2.5 mM), 0.5 μl of Taq polymerase, 1.0 μl (10 μM) of each primer, and 36.5 μl of sterile distilled water, was used to implement amplifications (Wang et al., 2015; Zhou et al., 2018). The PCR procedure was as follows: pre‐degeneration at 95°C for 5 min, degeneration at 94°C for 1 min, annealing at 54°C for 1 min, extension at 72°C for 1.5 min, with 35 cycles, and extension at 72°C for 10 min (Abe et al., 2008). It was stored at 10°C in the end of reaction process.

The PCR was produced in an ABI 3730 Automatic DNA Sequencer (Applied Biosystems) (Abe et al., 2008; Zhou et al., 2018). The received sequences were sent to GenBank of NCBI to seek similar sequences of type strain by using BLASTn (Wang et al., 2015). Multiple sequence alignment was carried out by using Clustal X for Windows. The evolution distance was calculated through a Kimura 2‐parameter of the MEGA 4.0 Soft. Neighbor‐joining method was used to establish phylogenetic trees.

### 2.5 Sensory evaluation

The sensory panel composed of seven panelists selected from 10 professional tea tasters, and the selection was based on evaluation performance of consistency and reliability (Qin et al., 2013). Sensory evaluation was based on five factors, including appearance (a), liquor color (b), aroma (c), taste (d), and infused leaves (e) according to China National Institute of Standardization (CNIS) GB/T 23776‐2018 (Gong et al., 2018). The evaluation procedures were as follows: A total of 100–150 g of tea samples were prepared for the evaluation of appearance; 3 g of samples was infused in 150 ml boiled water for 5 min; and the liquor color, aroma, taste, and infused leaves were evaluated after they were infused, respectively. Total score was estimated as follows:

Total score = 25%*(a) + 10%*(b) + 25%*(c) + 30%*(d) + 10%*(e).

### 2.6 Determination of chemical compositions

Moisture content and water extract content in each pu‐erh tea sample were developed according to CNIS GB/T 8304‐2013 (Wang et al., 2013) and GB/T 8305‐2013 (Zhou, Xu, Lu, Wang, & Sha, 2013), respectively. Contents of tea polyphenols (TP) and total free amino acids were determined by using the spectra photometric method based on FeSO_4_ and the ninhydrin assay (Liang, Zhang, & Lu, 2005), respectively. The main tea pigments, including theaflavins (TF), thearubigins (TR), and theabrownins (TB), were analyzed using the spectra photometric method described by Wang et al. (2011). Water‐soluble sugar (WSS) content was determined by visible spectrophotometer method with anthraquinone on 620 nm (Liang et al., 2005). Contents of GA and catechins, including C, EC, EGC, ECG, and EGCG, were determined by HPLC using an Agilent 1200 Series system with Agilent C_18_ Chromatogram column (250 mm × 4.6 mm, 5 μm) (Wang et al., 2016). Caffeine content was determined by Agilent 1200 HPLC equipment using Agilent C_18_ Chromatogram column (250 mm × 4.6 mm, 5 μm) with solvent A (100% acetonitrile) and solvent B (0.2% v/v acetic acid–water solution) as mobile phase (Tan et al., 2012).

### 2.7 Statistical analysis

Each pu‐erh tea sample was measured in three replications. All data are presented as mean value ± standard deviation (*SD*). The independent *t* test using SPSS 20.0 for Windows was carried out to determine whether the significant difference at *p* < .05 level or the highly significant difference at *p* < .01 level exists between JPT and GPT (control). Bivariate correlation analysis using SPSS 20.0 for Windows was carried out to determine effects of each dominant fungus on chemical composition after a long‐term storage.

## 3 RESULTS

### 3.1 Quality characteristic analysis of different pu‐erh teas

Quality characteristics were calculated by sensory evaluation based on five sensory factors. Significant difference analysis was carried out by using independent *t* test of SPSS 20.0. Results of sensory evaluation in GPT (control) and JPT are shown in Table 1. Due to both pu‐erh teas made from identical pu‐erh ripen tea (loose tea) after they were autoclaved, compressed, and dried, quality characteristics in appearance, liquor color, and infused leaves were similar with no significant differences (*p* > .05). However, due to the difference in storage environment, particularly the appearance of macroscopic golden cleistothecia, JPT had better aroma (*p* < .05) and taste (*p* < .01). Therefore, storage environment had significant (*p* < .05) impact on aroma and taste.

**TABLE 1:** Sensory evaluation and score of General pu‐erh tea (control) and Jinhua pu‐erh tea

Factors	Sensory evaluation		Score	
	GPT (control)	JPT	GPT (control)	JPT
Appearance (a)	Normal brick and well‐compressed	Obvious golden flower and well‐compressed	88 ± 1.73	87.3 ± 1.53
Liquor color (b)	Bright brownish red	Bright thick red	90.3 ± 1.15	91.0 ± 2.0
Aroma (c)	Stale pure flavor	Stale and harmonious arohid flavor	90.3 ± 1.15	94.7 ± 1.53*
Taste (d)	Stale mellow, pure, and thick	Stale mellow and smooth	87.0 ± 1.0	94.0 ± 1.0**
Infused leaves (e)	Fat, thick, and even	Fat, thick, and even	83.0 ± 1.0	83.6 ± 0.58
Total score	–	–	88.0 ± 0.72	91.2 ± 1.53*

Note

Score was present by mean value ± *SD* of three replications. Total score was the summation of products of factors and score coefficient. Total score = 25%*(a) + 10%*(b) + 25%*(c) + 30%*(d) + 10%*(e).

Abbreviations: GPT, General pu‐erh tea; JPT, Jinhua pu‐erh tea.

* There is significant difference in *p* < .05 level, and **a highly significant difference in *p* < .01 level using the independent *t* test of SPSS 20.0.

### 3.2 Differences in chemical compositions between GPT and JPT

To better understand the effect of storage environment on quality components, with GPT as control, main chemical components were determined in three replications. A comparison of isolated chemical components is presented in Table 2. The relative humid environment enhanced moisture content significantly (*p* < .05) in JPT. Compared with the control, the results showed significant (*p* < .05) difference of TF, TB, TP, and WSS contents and a highly significant difference of TR content in JPT, which illustrated that storage environment had significant (*p* < .05) impact on TF, TB, TP, and WSS contents, and a highly significant (*p* < .01) impact on TR, while it had no significant (*p* > .05) impacts on water extract content, GA, free amino acids, total catechins, and each catechin contents. The macroscopic golden cleistothecia in JPT enhanced WSS and TF contents significantly (*p* < .05) and TR content highly significantly (*p* < .01), while it decreased TP and TB contents significantly (*p* < .05). We speculated that filamentous fungi caused significant differences in given chemical compositions.

**TABLE 2:** A comparisons of the chemical components in General pu‐erh tea (control) and Jinhua pu‐erh tea

Samples	General pu‐erh tea (control)	Jinhua pu‐erh tea
Moisture content (%)	9.59 ± 0.48	10.55 ± 0.26*
Water extract content (%)	34.00 ± 1.51	36.33 ± 0.86
TF (mg/g)	3.63 ± 0.208	4.37 ± 0.208*
TR (mg/g)	36.43 ± 2.22	44.13 ± 1.21**
TB (mg/g)	131.7 ± 7.15	107.1 ± 11.13*
TP (mg/g)	85.73 ± 6.95	70.73 ± 2.15*
C (mg/g)	4.44 ± 1.14	4.14 ± 1.06
EC (mg/g)	13.7 ± 1.16	12.61 ± 0.98
EGC (mg/g)	14.99 ± 1.32	13.67 ± 0.91
ECG (mg/g)	10.33 ± 2.89	6.16 ± 1.11
EGCG (mg/g)	ND	ND
Total catechins (mg/g)	43.46 ± 4.18	36.6 ± 3.96
GA (mg/g)	12.81 ± 1.27	15.04 ± 1.36
Caffeine (mg/g)	38.42 ± 1.09	38.93 ± 0.76
WSS (mg/g)	33.40 ± 2.10	40.93 ± 2.17*
Free amino acids (mg/g)	6.43 ± 0.78	6.07 ± 1.17

Note

The content of total catechins was the summation of C, EC, EGC, ECG, and EGCG. All data were present by mean value ± *SD* of three replications.

Abbreviations: C, (+)‐catechin; EC, (−)‐epicatechin; ECG, (−)‐epicatechin 3‐*O*‐gallate; EGC, (−)‐epigallocatechin; EGCG, (−)‐epigallocatechin 3‐*O*‐gallate; GA, gallic acid; ND, not detected; TB, theabrownins; TF, theaflavins; TP, Tea polyphenols; TR, thearubigins; WSS, water‐soluble sugars.

* There is a significant difference at *p* < .05 levels, and **A highly significant difference at *p* < .01 levels using the independent *t* test of SPSS 20.0.

### 3.3 Biological identification of filamentous fungi

**FIGURE 2:**
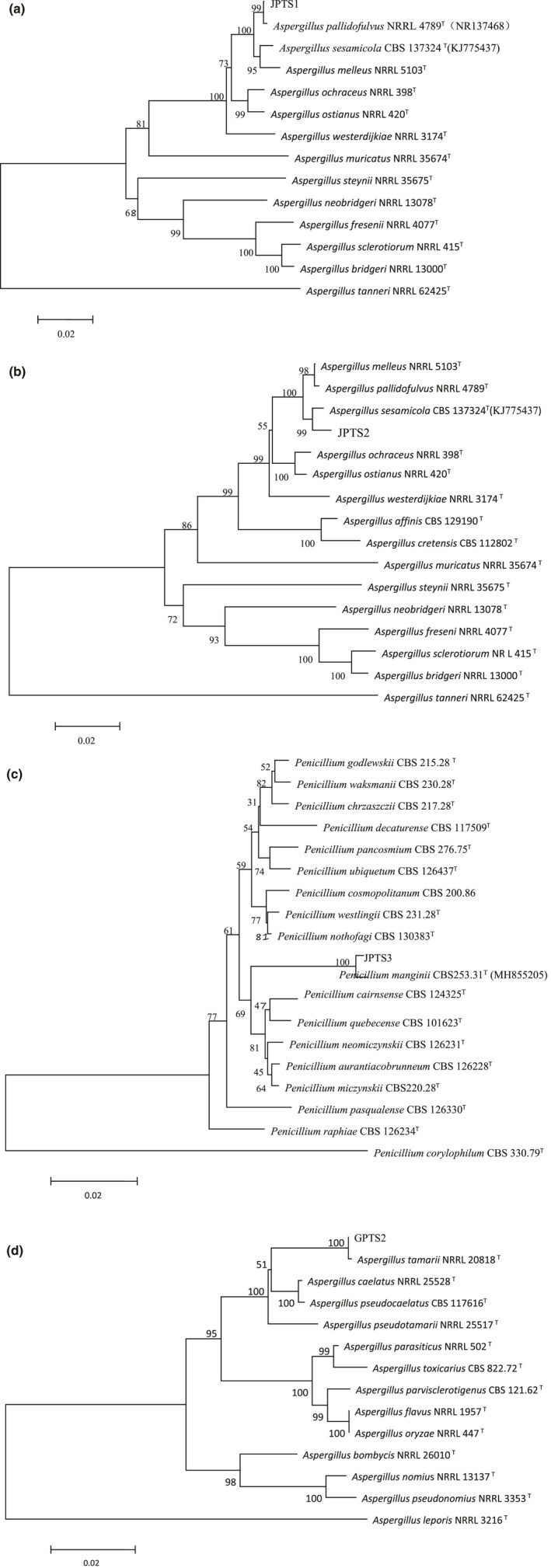
Phylogenetic trees of strains JPTS1 (a), JPTS2 (b), JPTS3 (c), and GTS2 (d) built based on the received sequences, respectively

In this paper, about nine different fungal strains were isolated from pu‐erh teas. Five filamentous fungi could be detected with a high abundance and named as GPTS1, JPTS1, JPTS2, JPTS3, and GPTS2, respectively, which were superior in numbers. Others were occasionally detected in pu‐erh teas, which belonged to the genera *Aspergillus*, *Penicillium*, and *Saccharomyces* according to colony morphological characteristics and conidia structures. The colony morphological characteristics of isolated filamentous fungi are shown in Table 3. Strain GPTS1 was identified as *A. niger* based on colony characteristics and conidial structure, which has been described in our previous study (Zhou et al., 2018). Strains JPTS1, JPTS2, and JPTS3 could generate yellow or golden cleistothecia in a particular state. PCR method was used to amplify the ITS, β‐tubulin, and calmodulin regions, respectively. Based on PCR‐amplified sequences (Figures S1–S4), phylogenetic trees of isolated filamentous fungi are built and shown in Figure 2. In the phylogram for *Aspergillus* species, strain JPTS1 was clustered with *A. pallidofulvus* and showed a 99.9% of identity to the tested strain NRRL4789 (NR137468), strain JPTS2 was clustered with *A. sesamicola* and showed a 99.8% of identity to the tested strain CBS137324 (KJ775437), and strain GPTS2 was clustered with *A. tamarii* and showed a 99.9% of identity to the tested strain NRRL20818 (AF433058). In the phylogram for *Penicillium* species, strain JPTS3 was identified as *P. manginii* and showed a 99.6% of identity to the tested strain CBS253.31 (MH855205). In conclusion, five filamentous fungi were isolated from pu‐erh teas and identified as *A. niger*, *A. pallidofulvus*, *A. sesamicola*, *A. tamarii*, and *P. manginii*, respectively. Among them, *A. pallidofulvus*, *A. sesamicola*, and *P. manginii* could generate macroscopic cleistothecia that were similar to the golden flowers appearing in JPT.

**TABLE 3:** Colony characteristics of filamentous fungi isolated from pu‐erh teas

Isolate	Shape	Surface	Color	Exudates	Cleistothecia
GPTS1	Circular	Rough	Black	None	None
JPTS1	Circular	Rough	Dark yellow colonies with white edges	Yellow sclerotium	Yellow
JPTS2	Irregular	Rough	Light yellow	Yellow sclerotium	Golden
JPTS3	Circular	Rough	Greyish‐green center with yellow patches	Red pigment	Yellow
GPTS2	Irregular	Rough	Hazel green with gray back	None	None

### 3.4 Distribution of fungal consortium in pu‐erh teas

**FIGURE 3:**
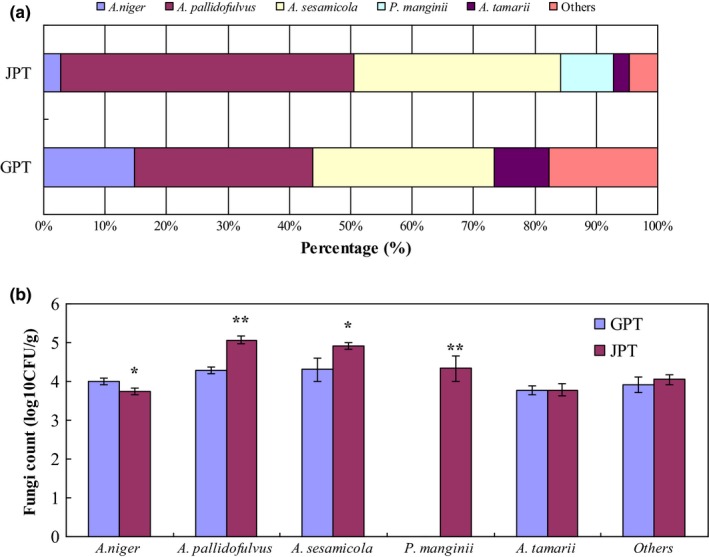
Distribution (a) and differences (b) of isolated filamentous fungi in pu‐erh tea type. GPT, General pu‐erh tea. JPT, Jinhua pu‐erh tea. About nine different kinds of fungal strains were isolated from pu‐erh tea samples based on PDA medium, and those colony‐forming units were calculated. Five filamentous fungi were isolated and identified. *Aspergillus pallidofulvus*, *Aspergillus sesamicola*, and *P. manginii* were dominant fungi in count and generated macroscopic golden cleistothecia after a long‐term storage in relative humid environment. Others were insignificant fungal strains in count, which belonged to the genera *Aspergillus*, *Penicillium*, and *Saccharomyces* according to the colony morphological characteristics and conidia structures. * indicates there is a significant difference at *p* < .05 level, and ** indicates a highly significant difference at *p* < .01 level using the independent *t* test of SPSS 20.0

The distribution and difference of fungal consortium are presented in Figure 3. The distribution of fungal consortium in JPT had obvious difference than in control (Figure 3a). All isolated filamentous fungi were detected in JPT, which occupied about 95.38% in total fungi count. However, *P. manginii* was not detected in GPT (control), and the isolated filamentous fungi occupied about 82.25% in count. Fungal flora in JPT had significant (*p* < .05) increase due to a high moisture content (Figure 3b). Particularly, *A. pallidofulvus* and *A. sesamicola* had a significant (*p* < .05) or a highly significant (*p* < .01) increase in JPT, respectively. Only *A. niger* had a significant (*p* < .05) decrease in JPT*.* The fungal flora, particularly the dominant fungi found in JPT including *A. pallidofulvus*, *A. sesamicola,* and *P. manginii*, caused significant differences of given chemical components in JPT after a long‐term storage. Correlation analysis was conducted later to investigate the relationship between each isolated filamentous fungus and chemical components, that is TF, TR, TB, TP, GA, and WSS, respectively.

### 3.5 Effects of isolated filamentous fungi on chemical compositions

In this paper, *A. niger*, *A. pallidofulvus*, *A. sesamicola*, *A. tamarii*, and *P. manginii* were isolated from pu‐erh teas. Particularly, *A. pallidofulvus*, *A. sesamicola*, and *P. manginii* were dominating fungi in JPT, which generate yellow or golden color cleistothecia in the storage. The relationships between each isolated filamentous fungus and chemical compound were evaluated by bivariate correlation analysis using SPSS 20.0 for Windows. The results are presented in Table 4. Table 4 shows that moisture content in pu‐erh tea had significantly (*p* < .05) positive correlations with *A. pallidofulvus* (*r* = .830) and *P. manginii* (*r* = .840), respectively. However, fungal community in pu‐erh tea storage had no significant (*p* > .05) correlations with water extract content, caffeine, free amino acids, and catechins including C, EC, EGC, ECG, and EGCG, respectively. The isolated filamentous fungi had positive or negative correlations with TP, TF, TR, TB, GA, and WSS. Specifically, *A. niger* had a significantly (*p* < .05) positive correlation with TP (*r* = .819), a significantly (*p* < .05) negative correlation with WSS (*r* = −.827), and a highly significantly (*p* < .01) negative correlation with TR (*r* = .931), respectively. *A. pallidofulvus* and *P. manginii* had highly significantly (*p* < .01) positive correlations with WSS (*r* = .921 and 0.923, respectively), which enhanced WSS content through degradation of water‐insoluble cellulose. Conversely, *A. sesamicola* had a highly significantly (*p* < .01) negative correlation with TB (*r* = −.929) and a significantly (*p* < .05) positive correlation with GA (*r* = .834), respectively, which caused significant (*p* < .05) decrease in TB and a slight increase in GA in JPT. *A. tamarii* had no significant (*p* > .05) impact on given chemical components. Therefore, *A. niger, A. pallidofulvus*, *A. sesamicola*, and *P. manginii* caused significant (*p* < .05) or highly significant (*p* < .01) differences of TP, TF, TR, TB, and WSS in JPT after a long‐term storage.

**TABLE 4:** Correlation coefficients of each isolate strain and chemical components

Indicators	*Aspergillus niger*	*Aspergillus pallidofulvus*	*Aspergillus sesamicola*	*Penicillium manginii*	*Aspergillus tamarii*	Others
Moisture content	0.544	0.830*	0.686	0.840*	0.230	−0.417
Water extract content	−0.784	0.785	0.568	0.810	−0.037	−0.229
TF	−0.762	0.834*	0.876*	0.851*	0.236	0.389
TR	−0.931**	0.851*	0.804*	0.806	−0.079	0.416
TB	0.561	−0.702	−0.929**	−0.722	−0.076	−0.163
TP	0.819*	−0.773	−0.801	−0.671	0.022	−0.693
C	−0.134	−0.273	−0.097	−0.501	0.396	−0.812*
EC	0.186	−0.448	−0.668	−0.595	−0.361	0.250
EGC	0.279	−0.592	−0.668	−0.711	−0.636	0.096
ECG	0.702	−0.636	−0.728	−0.563	0.029	−0.733
Total catechins	0.563	−0.519	−0.781	−0.446	0.173	−0.703
GA	−0.409	0.527	0.834*	0.482	−0.246	−0.018
Caffeine	0.059	0.243	0.469	−0.018	−0.154	−0.061
WSS	−0.827*	0.921**	0.772	0.923**	0.215	−0.016
Free amino acids	0.224	−0.463	−0.068	−0.576	−0.732	0.550

Abbreviations: C, (+)‐catechin; EC, (−)‐epicatechin; ECG, (−)‐epicatechin 3‐*O*‐gallate; EGC, (−)‐epigallocatechin; EGCG, (−)‐epigallocatechin 3‐*O*‐gallate; GA, gallic acid; TB, theabrownins; TF, theaflavins; TP, tea polyphenols; TR, thearubigins; WSS, water‐soluble sugars.

* A significant correlation at *p* < .05 level, **A highly significant correlation at *p* < .01 level.

## 4 DISCUSSION

Solid‐state fermentation plays an important role in the processing of pu‐erh tea (Abe et al., 2008). Fungi have profound impact on substance metabolisms and show correlation with quality formation of pu‐erh tea (Zhao et al., 2013, 2019). So far, *A. niger, Aspergillus tubingensis, Aspergillus fumigatus, Aspergillus acidus, Aspergillus awamori, A. tamarii, Aspergillus sydowii, Blastobotrys adeninivorans, Candida tropicalis, Fusarium graminearum, Pichia farinosa, Rasamsonia byssochlamydoides, Rasamsonia emersonii, Rasamsonia cylindrospora, Rhizomucor pusillus, Rhizomucor tauricus*, and *Thermomyces lanuginosus* have been detected in solid‐state fermentation of pu‐erh tea as dominant fungi (Wang et al., 2011; Zhang et al., 2016; Zhao et al., 2015; Zhou et al., 2018). In this study, five filamentous fungi were isolated from pu‐erh teas and identified as *A. niger*, *A. pallidofulvus*, *A. sesamicola*, *A. tamarii*, and *P. manginii*, respectively. The detection of *A. niger* and *Aspergillus tamari* in pu‐erh tea storage has a close connection with solid‐state fermentation of pu‐erh tea.

The strain generating yellow color cleistothecia is termed as the “golden flower fungus” (Mo, Zhu, & Chen, 2008; Mao, Wei, Teng, Huang, & Xia, 2017). The species within the genera *Aspergillus*, *Eurotium*, and *Penicillium* are main fungal taxa isolated from the postfermentation of dark tea (Mo et al., 2008). *Aspergillus cristatus* and *Eurotium cristatum* are golden flower fungi involved in the fermentation of Chinese fu‐zhuan tea (Ge et al., 2016; Zou et al., 2014). Numerous macroscopic yellow cleistothecia similar to that in other dark tea also could appear in pu‐erh tea after a long‐term storage, which was defined as JPT. With GPT made from the same pu‐erh ripen tea (loose tea) with identical processing as the control, through comparison of fungal consortium, *A. pallidofulvus*, *A. sesamicola*, and *P. manginii* were dominant fungi found in JPT and generate the appearance of macroscopic yellow cleistothecia after a long‐term storage, which had a significantly (*p* < .05) positive correlation with moisture content in pu‐erh tea cake.

The individual numbers of fungi in pu‐erh tea decreased significantly after solid‐state fermentation through autoclaving, compressing, and drying (Tian et al., 2013). The fungal flora is maintained at a relatively low level. Due to a long‐term storage for years, dominant fungi also had significant impact on chemical compositions during the storage, which caused significant (*p* < .05) differences of TF, TB, TP, and WSS contents, and a highly significant (*p* < .01) difference of TR between JPT and GPT (Table 2). Under effects of dominant fungi, TP could be oxidized to TF and TR, and TB had a significant decrease during the storage, which were different from the changes of chemical composition contents in solid‐state fermentation of pu‐erh tea (Chen, Cui, Li, Sheng, & Lv, 2013).

*Aspergillus* spp. including *A. tubingensis, A*. *fumigatus*, and *A. marvanovae* could convert TP to TB in solid‐state fermentation and submerged fermentation (Wang, Gong, Chisti, & Sirisansaneeyakul, 2014; Wang et al., 2015). *A. niger* and *A*. *fumigatus* could degrade ester catechins into nonester catechins and GA during solid‐state fermentation (Qin, Li, Tu, Ma, & Zhang, 2012). Additionally, *A. sydowii* could convert caffeine to theophylline and had significant impacts on WSS and free amino acid contents (Zhou, Ma, Ren, Xia, & Li, 2020; Zhou et al., 2019). Effects of each filamentous fungus on chemical compositions in the storage were investigated through the bivariate correlation analysis (Table 4). The results exhibited that the isolated dominant fungi had significant (*p* < .05) or highly significant (*p* < .01) impacts on given chemical compositions, including TP, TF, TR, TB, WSS, and GA after a long‐term storage.

## 5 CONCLUSIONS

Our present work describes fungal diversity and differences in chemical compositions between GPT and JPT to study effects of dominant fungi in pu‐erh tea storage. Due to the appearance of macroscopic yellow cleistothecia, JPT had better aroma (*p* < .05) and taste (*p* < .01) (Table 1) and had significant (*p* < .05) differences of TF, TB, TP, and WSS contents, and a highly significant (*p* < .01) difference of TR compared with the control (Table 2). *A. niger*, *A. pallidofulvus*, *A. sesamicola*, *A. tamarii*, and *P. manginii* were isolated from pu‐erh teas and identified. *A. pallidofulvus*, *A. sesamicola*, and *P. manginii* were dominant fungi and generated macroscopic yellow cleistothecia in JPT after a long‐term storage. The difference in fungal community should be ascribed to moisture content in tea cake and storage environment. Bivariate correlation analysis showed that the filamentous fungi had significantly positive or negative corrections with the differences of TF, TB, TP, WSS, and TR contents (Table 4). Particularly, *A. pallidofulvus* and *P. manginii* exhibited a highly significantly positive correlation (*p* < .01) with WSS and a significantly (*p* < .05) positive correlation with TF, while *A. sesamicola* showed a highly significantly negative correlation (*p* < .01) with TB and a significantly (*p* < .05) positive correlation with GA, respectively. Those results indicated that the isolated filamentous fungi had significant impact on given chemical compositions in the storage, which provides reference for the application of *A. pallidofulvus, A. sesamicola*, and *P. manginii* in tea.

## CONFLICT OF INTEREST

The authors declare that they have no conflict of interest.

## AUTHOR CONTRIBUTIONS

B. Zhou and C. Ma designed the research and wrote the paper. C. Ma and C. Zheng participated in the performance of the research. X. Liu conducted the statistical analysis. T. Xia contributed to the writing and data analysis. All authors read and approved the final manuscript.

## ETHICAL APPROVAL

The study did not involve any human or animal testing.

## Supporting information

Fig S1‐S4

## References

[fsn31543-bib-0001] Abe, M. , Takaoka, N. , Idemoto, Y. , Takagi, C. , Imai, T. , & Nakasaki, K. (2008). Characteristic fungi observed in the fermentation process for puer tea. International Journal of Food Microbiology, 124, 199–203. 18455823 10.1016/j.ijfoodmicro.2008.03.008

[fsn31543-bib-0002] Chen, C. S. , Chan, H. C. , Chang, Y. N. , Liu, B. L. , & Chen, Y. S. (2009). Effects of bacterial strains on sensory quality of pu‐erh tea in an improved pile‐fermentation process. Journal of Sensory Studies, 24, 534–553.

[fsn31543-bib-0003] Chen, H. X. , Cui, F. X. , Li, H. , Sheng, J. , & Lv, J. (2013). Metabolic changes during the pu‐erh tea pile‐fermentation revealed by a liquid chromatography tandem mass‐spectrometry‐based metabolomics approach. Journal of Food Science, 78(11), C1665–C1672. 24138293 10.1111/1750-3841.12288

[fsn31543-bib-0004] Chen, Y. S. , Liu, B. L. , & Chang, Y. N. (2010). Bioactivities and sensory evaluation of pu‐erh teas made from three tea leaves in an improved pile fermentation process. Journal of Bioscience and Bioengineering, 109(6), 557–563. 20471594 10.1016/j.jbiosc.2009.11.004

[fsn31543-bib-0005] Deng, Y. T. , Lin, S. Y. , Shyur, L. F. , & Lin, J. K. (2015). Pu‐erh tea polysaccharides decrease blood sugar by inhibition of α‐glucosidase activity *in vitro* and in mice. Food & Function, 6, 1539–1546. 25820466 10.1039/c4fo01025f

[fsn31543-bib-0006] Gao, L. , Bian, M. X. , Mi, R. F. , Hu, X. S. , & Wu, J. H. (2016). Quality identification and evaluation of pu‐erh tea of different grade levels and various ages through sensory evaluation and instrumental analysis. International Journal of Food Science and Technology, 51, 1338–1348.

[fsn31543-bib-0007] Ge, Y. , Wang, Y. , Liu, Y. , Tan, Y. , Ren, X. , Zhang, X. , … Liu, Z. (2016). Comparative genomic and transcriptomic analyses of the Fuzhuan brick tea‐fermentation fungus *Aspergillus cristatus* . BMC Genomics, 17, 428. 27267057 10.1186/s12864-016-2637-y PMC4895823

[fsn31543-bib-0008] Gong, S. Y. , Zhao, Y. X. , Lu, C. Y. , Liu, X. , Guo, Y. L. , Zhang, Y. B. , … Dang, Q. Y. (2018). Methodology for sensory evaluation of tea GB/T 23776. Beijing, China: China Standards Press.

[fsn31543-bib-0009] Lee, L. K. , & Foo, K. Y. (2013). Recent advances on the beneficial use and health implications of pu‐erh tea. Food Research International, 53, 619–628.

[fsn31543-bib-0010] Li, M. Y. , Xiao, Y. , Zhong, K. , Bai, J. R. , Wu, Y. P. , Zhang, J. Q. , & Gao, H. (2019). Characteristics and chemical compositions of Pinghu fuzhuan brick‐tea, a distinctive post‐fermentation tea in Sichuan province of China. International Journal of Food Properties, 22(1), 878–889.

[fsn31543-bib-0011] Liang, Y. R. , Zhang, L. Y. , & Lu, J. L. (2005). A study on chemical estimation of pu‐erh tea quality. Journal of the Science of Food and Agriculture, 85, 381–390.

[fsn31543-bib-0012] Lv, H. P. , Zhang, Y. J. , Lin, Z. , & Liang, Y. R. (2013). Processing and chemical constituents of Pu‐erh tea: A review. Food Research International, 53, 608–618.

[fsn31543-bib-0013] Lv, S. , Wu, Y. , Li, C. , Xu, Y. , Liu, L. , & Meng, Q. (2014). Comparative analysis of pu‐erh and fuzhuan teas by fully automatic headspace solid‐phase microextraction coupled with gas chromatography‐mass spectometry and chemometric methods. Journal of Agricultural and Food Chemistry, 62(8), 1810–1818. 24512533 10.1021/jf405237u

[fsn31543-bib-0014] Lyu, C. Y. , Chen, C. Y. , Ge, F. , Liu, D. Q. , Zhao, S. L. , & Chen, D. (2013). A preliminary metagenomic study of puer tea during pile fermentation. Journal of the Science of Food and Agriculture, 93, 3165–3174. 23553377 10.1002/jsfa.6149

[fsn31543-bib-0015] Mao, Y. , Wei, B. Y. , Teng, J. W. , Huang, L. , & Xia, N. (2017). Analyses of fungal community by Illumine Miseq platforms and characterization of *Eurotium* species on Liupao tea, a distinctive post‐fermented tea from China. Food Research International, 99, 641–649. 28784527 10.1016/j.foodres.2017.06.032

[fsn31543-bib-0016] Mo, H. , Zhu, Y. , & Chen, Z. (2008). Microbial fermented tea‐a potential source of natural food preservatives. Trends in Food Science & Technology, 19(3), 124–130.

[fsn31543-bib-0017] Nyambe, S. H. , & Williamson, G. (2016). Polyphenol‐ and fibre‐rich dried fruits with green tea attenuate starch‐derived postprandial blood glucose and insulin: A randomised, controlled, single‐blind, cross‐over intervention. British Journal of Nutrition, 116, 443–450. 27278405 10.1017/S0007114516002221

[fsn31543-bib-0018] Peng, C. X. , Wang, Q. P. , Liu, H. R. , Gao, B. , Shen, J. , & Gong, J. S. (2013). Effects of Zijuan pu‐erh tea theabrownin on metabolites in hyperlipidemic rat feces by Py‐GC/MS. Journal of Analytical and Applied Pyrolysis, 104, 226–233.

[fsn31543-bib-0019] Qin, J. H. , Li, N. , Tu, P. F. , Ma, Z. Z. , & Zhang, L. (2012). Change in tea polyphenol and purine alkaloid composition during solid‐state fungal fermentation of post‐fermented tea. Journal of Agricultural and Food Chemistry, 60, 1213–1217. 22239674 10.1021/jf204844g

[fsn31543-bib-0020] Qin, Z. , Pang, X. , Chen, D. , Cheng, H. , Hu, X. , & Wu, J. (2013). Evaluation of Chinese tea by the electronic nose and gas chromatography‐mass spectrometry: Correlation with sensory properties and classification according to grade level. Food Research International, 53, 864–874.

[fsn31543-bib-0021] Su, J. J. , Wang, X. Q. , Song, W. J. , Bai, X. L. , & Li, C. W. (2016). Reducing oxidative stress and hepatoprotective effect of water extracts from Pu‐erh tea on rats with high‐fat diet. Food Science and Human Wellness, 5, 199–206.

[fsn31543-bib-0022] Tan, H. P. , Xu, W. P. , Zhao, A. P. , Zhao, L. L. , Liu, M. D. , Tan, F. Y. , … Wang, Y. J. (2012). Determination of catechins and purine alkaloids in tea by high performance liquid chromatography. Analytical Letter, 45, 2530–2537.

[fsn31543-bib-0023] Tian, J. Q. , Zhu, Z. X. , Wu, B. , Wang, L. , & Liu, X. Z. (2013). Bacterial and fungal communities in pu er tea samples of different ages. Journal of Food Science, 78(8), M1249–M1256. 23957415 10.1111/1750-3841.12218

[fsn31543-bib-0024] Wang, C. , Lv, S. D. , Wu, Y. S. , Gao, X. M. , Li, J. B. , Zhang, W. R. , & Meng, Q. W. (2016). Oolong tea made from tea plants from different locations in Yunnan and Fujian, China showed similar aroma but different taste characteristics. SpringerPlus, 5, 576–591. 27247873 10.1186/s40064-016-2229-y PMC4864747

[fsn31543-bib-0025] Wang, J. , Zhou, W. L. , Xiao, W. H. , Sha, H. T. , Wang, H. B. , Xu, J. F. , & Lu, X. L. (2013). Tea‐determination of moisture content GB/T 8304. Beijing, China: China Standards Press.

[fsn31543-bib-0026] Wang, Q. P. , Gong, J. S. , Chisti, Y. , & Sirisansaneeyakul, S. (2014). Bioconversion of tea polyphenols to bioactive theabrownins by *Aspergillus fumigatus* . Biotechnology Letters, 36(12), 2515–2522. 25214210 10.1007/s10529-014-1632-0

[fsn31543-bib-0027] Wang, Q. P. , Gong, J. S. , Chisti, Y. , & Sirisansaneeyakul, S. (2015). Fungal isolates from a pu‐erh type tea fermentation and their ability to convert tea polyphenols to theabrownins. Journal of Food Science, 80, M809–M817. 25799937 10.1111/1750-3841.12831

[fsn31543-bib-0028] Wang, Q. P. , Peng, C. X. , & Gong, J. S. (2011). Effects of enzymatic action on the formation of theabrownin during solid state fermentation of Pu‐erh tea. Journal of the Science of Food and Agriculture, 91, 2412–2418. 21656777 10.1002/jsfa.4480

[fsn31543-bib-0029] Way, T. D. , Lin, H. Y. , Kuo, D. H. , Tsai, S. J. , Shieh, J. C. , Wu, J. C. , … Lin, J. K. (2009). Pu‐erh tea attenuates hyperlipogenesis and induces hepatoma cells growth arrest through activating AMP‐Activated Protein Kinase (AMPK) in human HepG2 cells. Journal of Agricultural and Food Chemistry, 57, 5257–5264. 19459711 10.1021/jf900730e

[fsn31543-bib-0030] Xu, A. , Wang, Y. , Wen, J. , Liu, P. , Liu, Z. , & Li, Z. (2011). Fungal community associated with fermentation and storage of fuzhuan brick tea. International Journal of Food Microbiology, 146(1), 14–22. 21345511 10.1016/j.ijfoodmicro.2011.01.024

[fsn31543-bib-0031] Xu, S. S. , Wang, J. J. , Wei, Y. M. , Deng, W. W. , Wan, X. C. , Bao, G. H. , … Ning, J. M. (2019). Metabolomics based on UHPLC‐Orbitrap‐MS and global natural product social molecular networking reveals effects of time scale and environment of storage on the metabolites and taste quality of raw pu‐erh tea. Journal of Agricultural and Food Chemistry, 67(43), 12084–12093. 31560531 10.1021/acs.jafc.9b05314

[fsn31543-bib-0032] Zhang, L. , Li, N. , Ma, Z. Z. , & Tu, P. F. (2011). Comparison of the chemical constituents of aged pu‐erh tea, ripen pu‐erh tea, and other teas using HPLC‐DAD‐ESI‐MS^n^ . Journal of Agricultural and Food Chemistry, 59, 8754–8760. 21793506 10.1021/jf2015733

[fsn31543-bib-0033] Zhang, W. , Yang, R. J. , Fang, W. J. , Yan, L. , Lu, J. , Sheng, J. , & Lv, J. (2016). Characterization of thermophilic fungal community associated with pile fermentation of pu‐erh tea. International Journal of Food Microbiology, 227, 29–33. 27046629 10.1016/j.ijfoodmicro.2016.03.025

[fsn31543-bib-0034] Zhao, M. , Su, X. Q. , Nian, B. , Chen, L. J. , Zhang, D. L. , Duan, S. M. , … Ma, Y. (2019). Integrated meta‐omics approaches to understand the microbiome of spontaneous fermentation of traditional Chinese pu‐erh tea. Msystems, 4, e00680. 31744906 10.1128/mSystems.00680-19 PMC6867877

[fsn31543-bib-0035] Zhao, M. , Xiao, W. , Ma, Y. , Sun, T. T. , Yuan, W. X. , Na, T. , … Cui, X. L. (2013). Structure and dynamics of the bacterial communities in fermentation of the traditional Chinese post‐fermented pu‐erh tea revealed by 16S RNA gene clone library. World Journal of Microbiology & Biotechnology, 29, 1877–1884. 23591759 10.1007/s11274-013-1351-z

[fsn31543-bib-0036] Zhao, M. , Zhang, D. L. , Su, X. Q. , Duan, S. M. , Wan, J. Q. , Yuan, W.‐X. , … Pan, Y.‐H. (2015). An integrated metagenomics/metaproteomics investigation of the microbial communities and enzymes in solid‐state fermentation of pu‐erh tea. Scientific Reports, 5, 10117. 25974221 10.1038/srep10117 PMC4431464

[fsn31543-bib-0037] Zhou, B. X. , Ma, C. Q. , Ren, X. Y. , Xia, T. , & Li, X. H. (2020). LC‐MS/MS‐based metabolomic analysis of caffeine‐degrading fungus *Aspergillus sydowii* during tea fermentation. Journal of Food Science, 85(2), 477–485. 31905425 10.1111/1750-3841.15015

[fsn31543-bib-0038] Zhou, B. X. , Ma, C. Q. , Ren, X. Y. , Xia, T. , Li, X. H. , & Wu, Y. (2019). Production of theophylline via aerobic fermentation of pu‐erh tea using tea‐derived fungi. BMC Microbiology, 19, 261. 31771506 10.1186/s12866-019-1640-2 PMC6878699

[fsn31543-bib-0039] Zhou, B. X. , Ma, C. Q. , Wang, H. Z. , & Xia, T. (2018). Biodegradation of caffeine by whole cells of tea‐derived fungi *Aspergillues sydowi*i, *Aspergillus niger* and optimization for caffeine degradation. BMC Microbiology, 18, 53 10.1186/s12866-018-1194-8 29866035 PMC5987490

[fsn31543-bib-0040] Zhou, W. L. , Xu, J. F. , Lu, X. L. , Wang, J. , & Sha, H. T. (2013). Tea‐determination of water extracts content GB/T 8305. Beijing, China: China Standards Press.

[fsn31543-bib-0041] Zou, X. , Li, Y. , Zhang, X. , Li, Q. , Liu, X. , Huang, Y. , … Tang, J. T. (2014). A new prenylated indole diketopiperazine alkaloids from *Eurotium cristatum* . Molecules, 19(11), 17839–17847. 25372398 10.3390/molecules191117839 PMC6271712

