# Supplementary material for: Correlation analysis between filamentous fungi and chemical compositions in a pu‐erh type tea after a long‐term storage
Source: Food Sci Nutr. 2020 Apr 13;8(5):2501–11. doi: 10.1002/fsn3.1543 (PMC7215201; doi:10.1002/fsn3.1543)
Supplement: Supplementary file 1 — Fig S1‐S4 [file FSN3-8-2501-s001.doc]

ITS sequence of strain JPTS1 (541 bp)：

1 TCGGGGCCACCTCCCACCCGTGTATACCGTACCTTGTTGCTTCGGCGAGC 50

51 CCGCCCCCTTTTTCTTAGGGGGGCACAGCGCTCGCCGGAGACACCAACGT 100

101 GAACACTGTCTGAAGTTTTGTCGTCTGAGTCGATTGTATCGCAATCAGTT 150

151 AAAACTTTCAACAATGGATCTCTTGGTTCCGGCATCGATGAAGAACGCAG 200

201 CGAAATGCGATAATTAATGTGAATTGCAGAATTCAGTGAATCATCGAGTC 250

251 TTTGAACGCACATTGCACCCCCTGGTATTCCGGGGGGTATGCCTGTCCGA 300

301 GCGTCATTGCTGCCCTCAAGCACGGCTTGTGTGTTGGGTCGTCGTCCCCC 350

351CCGGGGGACGGGCCCGAAAGGCAGCGGCGGCACCGCGTCCGGTCCTCGAG 400

401 CGTATGGGGCTTTGTCACACCGCTCTTGTAGGCCCGGCCGGCTGCTGGCC 450

451 GACGCTGAAAAGCAACCAACTATTTCTCCAGGTTGACCTCGGATCAGGTA 500

501 GGGATACCCGCTGAACTTAAGCATATCAATAAGCGGAGGAA 541

β-tubulin sequence of strain JPT-1 (516 bp):

1 TCATCTTCGATACCTTGTGACCTATGACTCTCAATCCTTGATACTTGATA 50

51 CTTGTTTACTGATAGGTGAATAGGCAAAACATCTCTGGCGAGCACGGCCT 100

101 TGATGGCGCCGGTGTGTAAGTACATCCCGCGTTTACACCTATCGAAATCA 150

151 GAATCGACGAGAGAAGAAAAGAAAGAAATGATCATGGTGGGATTGATTGT 200

201 CTGATGGGATGAACAGTTACAATGGCTCCTCCGACCTTCAGCTGGAGCGC 250

251 ATGAACGTCTACTTCAACGAGGTTCGTTGCCCGAAAATTTTCTATCTCCT 300

301 TTCGCCGATCCGAAACGCCCCGTACAAGGCTCTAACCCACGCTTTCTTCA 350

351 TCTTCTAGGCTTCCGGTGGCAAGTATGTTCCCCGTGCCGTTCTGGTCGAT 400

401 CTTGAGCCCGGTACCATGGACGCTGTCCGTGCCGGTCCCTTCGGTCAGCT 450

451 TTTCCGCCCCGACAACTTCGTCTTCGGCCAGTCTGGTGCCGGTAACAACT 500

501 GGGCCAAGGGTCACTA 516

Calmodulin sequence of strain JPTS1 (765 bp):

1 TTCGTAGTGTTCCATTCTTCCTGATGTAATGATGGGGAAACGAAGCGATC 50

51 TTGCTTTGTTTTCTACGACTTCAGCCTTATGGGAATATTCCAAGCTCACA 100

101 TGAGATTTTGCCTCCTCACAGGACAAGGATGGCGATGGTTAGTGCTATTC 150

151 CGGTTTCCCTTCCAATTCATCGACTCACGCGACCGGCTATTTTCCGTCGA 200

201 TATGGAATTTTTTTCACCCTGTTGCTTCCGGCGACCGATTTGCGACCAGG 250

251 ACGCTAATTTGTGAATTCACGCTGCAGGCCAGATCACCACCAAGGAGTTG 300

301 GGCACTGTTATGCGCTCGCTGGGCCAGAACCCCTCCGAGTCTGAGTTGCA 350

351 GGACATGATCAACGAGGTTGACGCCGACAACAATGGCACCATCGATTTCC 400

401 CCGGTATGCGATGATGGACACACCGATATCTGGAAAGGGAGACAATCCTG 450

451 AAACTCAGCTGCTAACCTCACGCAGAGTTCCTGACGATGATGGCCCGAAA 500

501 GATGAAGGATACCGATTCTGAGGAGGAAATCCGGGAAGCTTTCAAGGTCT 550

551 TCGATCGCGATAACAACGGTTTCATCTCGGCCGCGGAGCTGCGCCACGTC 600

601 ATGACCTCCATCGGCGAGAAGCTCACCGACGACGAGGTCGATGAGATGAT 650

651 CCGTGAGGCGGACCAGGATGGCGACGGCCGGATTGACTGTACGTTGTGAC 700

701 CTTGGGATGCCCTTTCTGAACCCAAACTAATGAACTGCTCCAGACAACGA 750

751 GTTCGTCCAACTTAT 765

**Additional File 1: Figure S1.** The received sequences of strain JPTS1.

ITS sequence of strain JPTS2 (532 bp)：

1 CCTCCCACCCGTGTATACCGTACCTTGTTGCTTCGGCGAGCCCGCCCCCT 50

51 TTTTCTTAGGGGGGCACAGCGCTCGCCGGAGACACCAACGTGAACACTGT 100

101 CTGAAGTTTTGTCGTCTGAGTCGATTGTATCGCAATCAGTTAAAACTTTC 150

151 AACAATGGATCTCTTGGTTCCGGCATCGATGAAGAACGCAGCGAAATGCG 200

201 ATAATTAATGTGAATTGCAGAATTCAGTGAATCATCGAGTCTTTGAACGC 250

251 ACATTGCACCCCCTGGTATTCCGGGGGGTATGCCTGTCCGAGCGTCATTG 300

301 CTGCCCTCAAGCACGGCTTGTGTGTTGGGTCGTCGTCCCCCCCGGGGGAC 350

351 GGGCCCGAAAGGCAGCGGCGGCACCGCGTCCGGTCCTCGAGCGTATGGGG 400

401 CTTTGTCACACCGCTCTTGTAGGCCCGGCCGGCTGCTGGCCGACGCTGAA 450

451 AAGCAACCAACTATTTCTCCAGGTTGACCTCGGATCAGGTAGGGATACCC 500

501 GCTGAACTTAAGCATATCAATAAGCGGAGGAA 532

β-tubulin sequence of strain JPTS2 (515 bp):

1 CATCTTCGATACCTTGTGACCTATGACTCTCAATCCTTGATACTTGATAC 50

51 TTGTTTACTGATAGGTGAATAGGCAAAACATCTCTGGCGAGCACGGCCTT 100

101 GATGGCGCCGGTGTGTAAGTACATCCCGCGTTTACACCTATCGAAATCAG 150

151 AATCGACGAGAGAAGAAAAGAAAGAAATGATCATGGTGGGATTGATTGTC 200

201 TGATGGGATGAACAGTTACAATGGCTCCTCCGACCTTCAGCTGGAGCGCA 250

251 TGAACGTCTACTTCAACGAGGTTCGTTGCCCGAAAATTTTCTATCTCCTT 300

301 TCGCCGATCCGAAACGCCCCGTACAAGGCTCTAACCCACGCTTTCTTCAT 350

351 CTTCTAGGCTTCCGGTGGCAAGTATGTTCCCCGTGCCGTTCTGGTCGATC 400

401 TTGAGCCCGGTACCATGGACGCTGTCCGTGCCGGTCCCTTCGGTCAGCTT 450

451 TTCCGCCCCGACAACTTCGTCTTCGGCCAGTCTGGTGCCGGTAACAACTG 500

501 GGCCAAGGGTCACTA 515

Calmodulin sequence of strain JPTS2 (757 bp):

1 GTGTTCCATTCTTCCTGATGTAATGATGGGGAAACGAAGCGATCTTGCTT 50

51 TGTTTTCTACGACTTCAGCCTTATGGGAATATTCCAAGCTCACATGAGAT 100

101 TTTGCCTCCTCACAGGACAAGGATGGCGATGGTTAGTGCTATTCCGGTTT 150

151 CCCTTCCAATTCATCGACTCACGCGACCGGCTATTTTCCGTCGATATGGA 200

201 ATTTTTTTCACCCTGTTGCTTCCGGCGACCGATTTGCGACCAGGACGCTA 250

251 ATTTGTGAATTCACGCTGCAGGCCAGATCACCACCAAGGAGTTGGGCACT 300

301 GTTATGCGCTCGCTGGGCCAGAACCCCTCCGAGTCTGAGTTGCAGGACAT 350

351 GATCAACGAGGTTGACGCCGACAACAATGGCACCATCGATTTCCCCGGTA 400

401 TGCGATGATGGACACACCGATATCTGGAAAGGGAGACAATCCTGAAACTC 450

451 AGCTGCTAACCTCACGCAGAGTTCCTGACGATGATGGCCCGAAAGATGAA 500

501 GGATACCGATTCTGAGGAGGAAATCCGGGAAGCTTTCAAGGTCTTCGATC 550

551 GCGATAACAACGGTTTCATCTCGGCCGCGGAGCTGCGCCACGTCATGACC 600

601 TCCATCGGCGAGAAGCTCACCGACGACGAGGTCGATGAGATGATCCGTGA 650

651 GGCGGACCAGGATGGCGACGGCCGGATTGACTGTACGTTGTGACCTTGGG 700

701 ATGCCCTTTCTGAACCCAAACTAATGAACTGCTCCAGACAACGAGTTCAT 750

751 CCAACTC 757

**Additional File 1: Figure S2.** The received sequences of strain JPTS2.

ITS sequence of strain JPTS3 (525 bp)：

1 ACCTCCCACCCGTGTTTAACGAACCGTGTTGCTTCGGCGGGCCCGCCTCA 50

51 CGGCCGCCGGGGGGCATCCGCCCCCGGGCCCGCGCCCGCCGAAGCCCCCT 100

101 GTGAACGCTGTCTGAAGTATGCAGTCTGAGACAATTATTCAATTAATTAA 150

151 AACTTTCAACAACGGATCTCTTGGTTCCGGCATCGATGAAGAACGCAGCG 200

201 AAATGCGATAACTAATGTGAATTGCAGAATTCAGTGAATCATCGAGTCTT 250

251 TGAACGCACATTGCGCCCTCTGGTATTCCGGAGGGCATGCCTGTCCGAGC 300

301 GTCATTGCTGCCCTCCAGCCCGGCTGGTGTGTTGGGCCCCCGCCCCCCTT 350

351CCCGGGGGGGCGGGCCCGAAAGGCAGCGGCGGCACCGCGTCCGGTCCTCG 400

401 AGCGTATGGGGCTTTGTCACCCGCTCTTGCAGGCCCGGCCGGCGCCAGCC 450

451 GACCCCCTCAATCTATTTTTTCAGGTTGACCTCGGATCAGGTAGGGATAC 500

501 CCGCTGAACTTAAGCATATCAATAG 525

β-tubulin sequence of strain JPTS3 (420 bp):

1 GGCACTCGAAGCAATATACTAACCAATTTTACAGGCAAACCATTGCCGGT 50

51 GAGCACGGCCTTGATGGCGATGGACAGTGAGTTCTTTAGACAACCTTTTG 100

101 ATTTTCGAGAATGGCGGTCTGATATTTTTGGGCAGGTACAACGGTACTTC 150

151 CGACCTCCAGCTGGAGCGCATGAACGTCTACTTCACCGAAGTAAGGGATC 200

201 TCGACATCAATTCTACTGACGATTCTCATTCTGACTGGTCGTTTCTTTTC 250

251 TCTCCAATAGGCTTCCGGTGACAAGTATGTTCCCCGTGCCGTTCTGGTCG 300

301 ATCTGGAGCCCGGTACCATGGACGCTGTCCGTGCCGGTCCTTTCGGCAAG 350

351 CTCTTCCGCCCCGACAACTTCGTCTTCGGTCAGTCTGGTGCTGGTAACAA 400

401 CTGGGCCAAGGGTCACTAAC 420

**Additional File 1: Figure S3.** The received sequences of strain JPTS3.

ITS sequence of strain GPTS2 (532 bp):

1CCTCCCACCCGTGTTTACTGTAACCTTAGTTGCTTCGGCGGGCCCGCCTT50

51TAAGGCCGCCGGGGGGCATCAGCCCCCGGGCCCGCGCCCGCCGGAGACAC100

101CACGAACTCTGTCTGATCTAGTGAAGTCTGAGTTGATTGTATCGCAATCA150

151GTTAAAACTTTCAACAATGGATCTCTTGGTTCCGGCATCGATGAAGAACG200

201CAGCGAAATGCGATAACTAGTGTGAATTGCAGAATTCCGTGAATCATCGA250

251GTCTTTGAACGCACATTGCGCCCCCTGGTATTCCGGGGGGCATGCCTGTC300

301CGAGCGTCATTGCTGCCCATCAAGCACGGCTTGTGTGTTGGGTCGTCGTC350

351CCCTCTTCGGGGGGGACGGGCCCCAAAGGCAGCGGCGGCACCGCGTCCGA400

401TCCTCGAGCGTATGGGGCTTTGTCACCCGCTCTGTAGGCCCGGCCGGCGC450

451TTGCCGAACGCAAAACAACCATTCTTTCCAGGTTGACCTCGGATCAGGTA500

501GGGATACCCGCTGAACTTAAGCATATCAATAA532

β-tubulin sequence of strain GPTS27 (476 bp):

1CAAGGAACTGCACAGAAGCATGAACTCAGATGTGCCCTACTGTGTCTGCC50

51ACGTGTTTGCTAACATCTTTGCAGGCAGACCATCTCTGGCGAGCACGGCC100

101TTGACGGCTCCGGTGTGTAAGTACAATCCGTGTACACCTCGAACGAACGA150

151CAACCAGATGGCATTGGAAGAGTTGGAATGGGTCTGACGGGAAGGATAGT200

201TACAATGGCTCCTCCGATCTCCAGCTGGAGCGTATGAACGTCTACTTCAA250

251CGAGGTGCGTACCTCACATTTTTCAGCCTCTTTGACAACGCTTTGCAAGT300

301CCTGACCGCTTCTCCAGGCCAGCGGAAACAAGTATGTCCCTCGTGCCGTC350

351CTTGTCGATCTTGAGCCCGGTACCATGGACGCCGTCCGTGCCGGTCCCTT400

401CGGTCAGCTGTTCCGTCCCGACAACTTCGTTTTCGGCCAGTCCGGTGCTG450

451GTAACAACTGGGCCAAGGGTCACTAC476

Calmodulin sequence of strain GPTS2 (715 bp):

1GTCATGAACGTCGTTCGCGAAAATCGGCTTTGTGAGTAGACTTTATTTGA50

51ACACAAGCTGACTGGGCTTCTCTTGGGTTTCCTATAGGACAAGGACGGTG100

101ATGGTTAGTACAGTCTCTTTCATTCCGTCTCCCTTCAAATGCGACCAGTA150

151TCTTTTAGCCGGCATAGTTTTATCCATTTTCTGTTCGATCGGCTGAAGTC200

201TTTGGCATTGATGGATTGACTTGATATGCAGGCCAGATCACCACCAAGGA250

251GTTGGGCACTGTCATGCGCTCTCTGGGCCAAAACCCCTCTGAGTCGGAAC300

301TCCAGGACATGATTAACGAAGTTGACGCCGATAACAATGGCACCATTGAC350

351TTTCCTGGTACGAGAGGGCTTCCGTACATTTTACAAATAAAATAGCTGTT400

401AATGTTCAACCAGAGTTCCTCACGATGATGGCGAGAAAGATGAAGGATAC450

451CGACTCTGAGGAGGAGATCCGGGAGGCTTTCAAGGTTTTCGACCGCGATA500

501ACAACGGTTTCATCTCCGCTGCCGAATTGCGCCACGTCATGACCTCCATC550

551GGCGAGAAGCTTACCGATGACGAAGTTGATGAGATGATCCGCGAGGCGGA600

601TCAGGATGGTGACGGTCGGATCGACTGTATGTTTCGAGAAGCCCTCCCAC650

651ACACACCTATTGCGGCTGTGAAACCGGTGATACTGATCGATTTTAGACAA700

701CGAGTTCGTCCAACT715

**Additional File 1: Figure S4.** The received sequences of strain GPTS2.
